# Rare central nervous system manifestation of granulomatosis with polyangiitis in a 12-year-old child: A case report

**DOI:** 10.1016/j.radcr.2023.07.077

**Published:** 2023-08-10

**Authors:** Neda Azin, Ali Hajihashemi, Mahsa Geravandi

**Affiliations:** a Department of Radiology, Imam Hussein Children Hospital, Isfahan University of Medical Sciences, Isfahan, Iran; b Department of Radiology, Isfahan University of Medical Sciences, Isfahan, Iran

**Keywords:** Wegener's granulomatosis, Granulomatosis with polyangiitis, Pediatrics, Posterior reversible encephalopathy syndrome

## Abstract

To share a unique case of granulomatosis with polyangiitis (GPA) identified in a child with CNS involvement, specifically PRES (posterior reversible encephalopathy syndrome). Discuss this uncommon manifestation's clinical characteristics, diagnostic process, and treatment. We are currently discussing a 12-year-old female patient who presented with a chronic cough, shortness of breath, and a new-onset fever. Upon further examination, the patient was diagnosed with GPA, confirmed through positive cytoplasmic antineutrophil cytoplasmic antibodies (C-ANCA), a renal biopsy, and multiple lung cavitary lesions. During her hospitalization, the patient also experienced neurological symptoms, including a severe headache, blurred vision, loss of consciousness, and an abnormal neurological exam, which led to brain MR imaging. The imaging revealed evidence of small vessel vasculitis with confluent T2 hyper signal intensity of gray-white matter junctions in both parietooccipital and frontal lobes containing hemorrhagic components, suggesting Posterior reversible encephalopathy syndrome. This case of Wegener's granulomatosis is noteworthy due to its occurrence in a pediatric patient with CNS involvement, specifically (posterior reversible encephalopathy syndrome). This event highlights the importance of recognizing that autoimmune disorders can present infrequently in young patients. Diagnosing Wegener's granulomatosis can be challenging, particularly when the CNS is affected. However, when appropriate treatment is initiated promptly, favorable outcomes can be achieved, as evidenced by the patient's improved condition with the prednisolone, captopril, and Rituximab treatment plan. Further research is necessary to understand better the underlying pathophysiology and optimal management of CNS involvement in GPA, particularly in the pediatric population.

## Background

Granulomatosis with polyangiitis (GPA) is a rare disorder affecting small and medium-sized blood arteries in children [1,2]. It is characterized by necrotizing granulomatous vasculitis with positive C-ANCA defining features. GPA affects multiple organ systems, with pulmonary and renal manifestations occurring at 90% and 80%, respectively. Pediatric presentations vary, with common symptoms in children including constitutional, pulmonary, ear, nose, throat, and renal manifestations [3], [4], [5]. Central nervous system involvement is uncommon, reported in 7%-11% of patients. Common symptoms includemultiple cranial neuropathies, cerebrovascular events, pachymeningitis, seizures, and PRES (posterior reversible encephalopathy syndrome), which may affect children more than adults due to limited brain autoregulation [[6], [7], [8]].

## Case presentation

A 12-year-old girl presented to the children's hospital with a 2-month history of coughing and shortness of breath. Her parents explained that the symptoms started suddenly, and she had previously been treated with antibiotics and bronchodilators, although her symptoms persisted. Her condition worsened progressively, and her parents denied hemoptysis, GI (gastrointestinal) symptoms, night sweats, or weight loss except for a low-grade fever the night before admission. The birth history was uneventful, and the developmental milestones were normal. Before her illness, she had no notable medical history and was up-to-date on her immunizations. Additionally, there was no significant family history of autoimmune diseases or respiratory disorders.

On the physical exam, she appeared sick with mild respiratory distress. Upon admission, the vital signs were as follows: blood pressure was 130/100 mm Hg, temperature was 39°C, heart rate was 80 beats per minute, her respiratory rate was 18 breaths per minute, and oxygen saturation was 90% in room air. Moreover, bilateral rales were observed during the pulmonary examination without noticeable crackling. A cranial nerve examination demonstrated no abnormal findings. The motor examination was normal. A neurologic examination showed normal gait and reflexes.

Moreover, there was no evidence of oral ulcers, skin lesions, nasal discharge, lymphadenopathy, or arthritis. Additionally, the ophthalmology examination appeared normal, along with other systems examinations.

Lab data revealed an elevated erythrocyte sedimentation rate of 40 mm/h and a c-reactive protein of 50 mg/L. There was neutrophil-dominant leukocytosis (white blood cells: 25000/mm^3^ and neutrophil count: 20,100/mm^3^) and anemia (Hb: 9.2 g/dL). The kidney function test illustrated that serum creatinine levels were increased (2.5g/dL) with normal blood urea nitrogen.

Moreover, urinalysis showed hematuria (Blood 2+ and 50-60 red blood cells per microscopic high-power field).

The results of normal in the evaluation of the complement level, Wright, and 2-mercaptoethanol tests, as well as the systemic lupus erythematosus autoantibodies panel, perinuclear antineutrophil cytoplasmic antibody, and immunoglobulin level tests except cytoplasmic antineutrophil cytoplasmic antibodies.

**Fig. 1:**
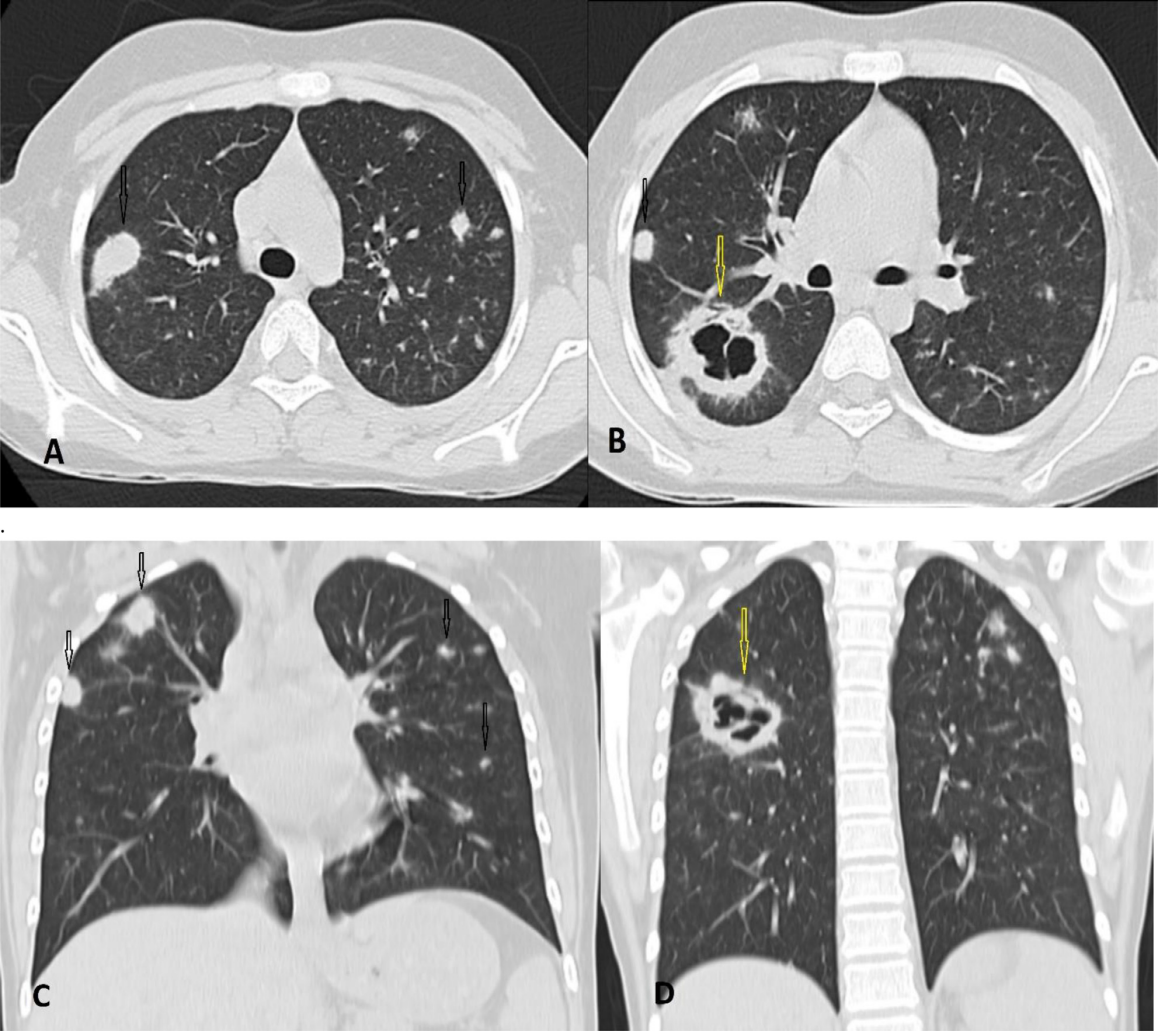
Axial (A, B) and coronal (C, D) lung view of a thoracic CT scan without contrast: Multiple pulmonary nodules and masses in both lungs, affecting the upper and lower lobes, are present. (Black arrows) Some of these masses have central cavitation (yellow arrows). Additionally, some nodules have peripheral ground glass attenuated noted.

**Fig. 2:**
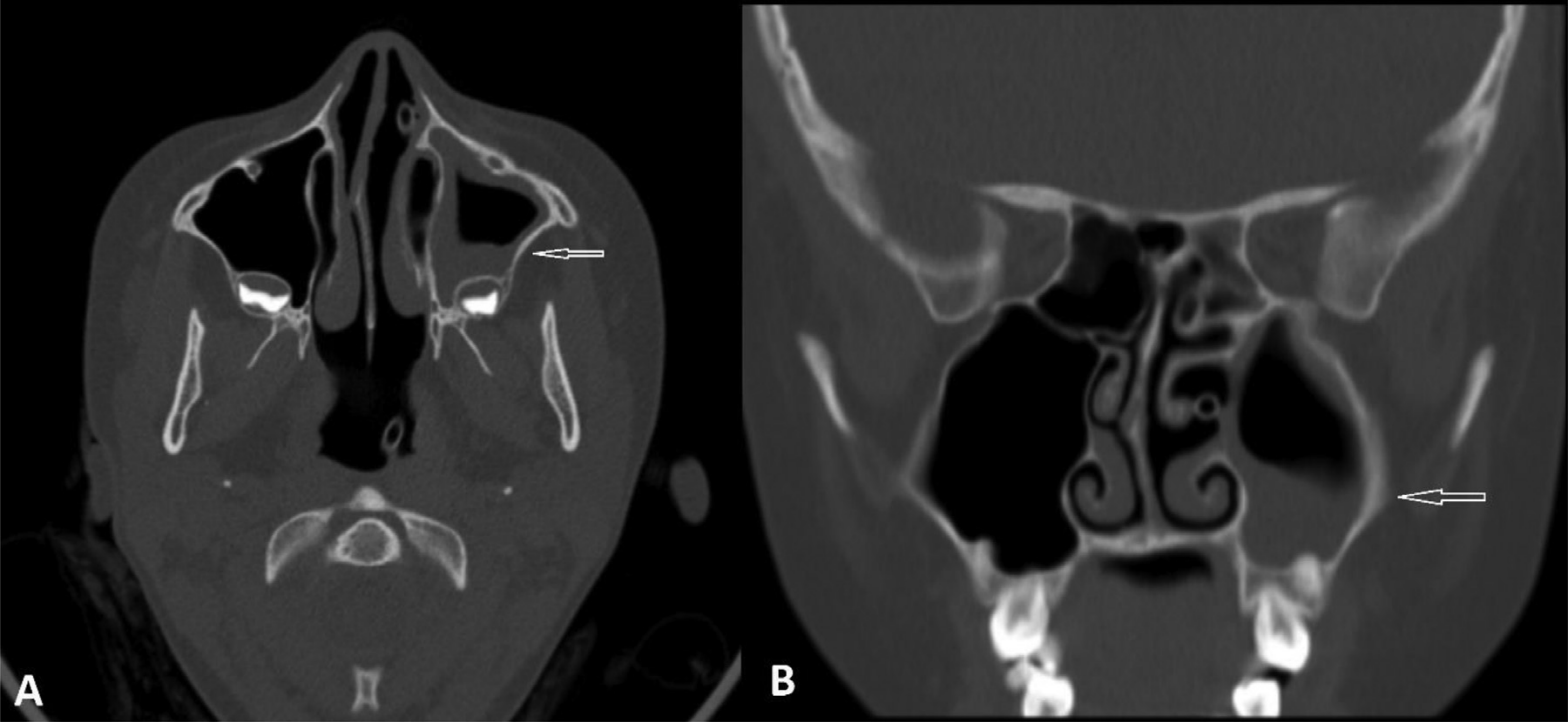
Axial (A) and coronal (B) paranasal sinuses CT scan without contrast: evidence of left maxillary sinus mucosal thickening is noted (arrows).

As a result of the patient's cough and difficulty breathing, chest and paranasal sinuses, computed tomography (CT) scans were performed without contrast. The results revealed the presence of multiple pulmonary vessel-related nodules and masses scattered throughout both lungs without predilection, some of which showed central cavitation and thick irregular walls (Fig. 1). No signs of tracheobronchial or pleural/mediastinal involvement were observed. The CT scan of the peripheral nervous system (PNS) has indicated the presence of mucosal thickening in the left maxillary sinus, which is suggestive of sinusitis. There is no visible bone erosion or remodeling observed (Fig. 2). Furthermore, the ultrasonography of the kidneys indicated enlarged and echogenic kidneys with normal blood flow and no signs of hydronephrosis.

Based on the patient's clinical presentation, lab results, and chest CT scan, the initial diagnosis was vasculitis, specifically GPA. A renal biopsy was performed in order to diagnosis confirmation. According to the histopathology evaluation, the final diagnosis was granulomatosis with polyangiitis.

The patient started induction therapy on high-dose intravenous methylprednisolone (40 mg/d) for 4 weeks and intravenous rituximab (375 mg/m^2^ once a week for 4 weeks) with antibiotics.

Following a week-long hospital stay, the patient had shown improvement in her respiratory symptoms. Nevertheless, she experienced the unexpected onset of a severe headache, blurred vision, and loss of consciousness. Notably, the patient had not previously reported any history of headaches, visual disturbances, or seizures. She had high blood pressure, measured at 150/110 mmHg, and was also experiencing tachycardia. During a neurologic examination, papilledema was detected, but no focal neurological deficits were found.

**Fig. 3:**
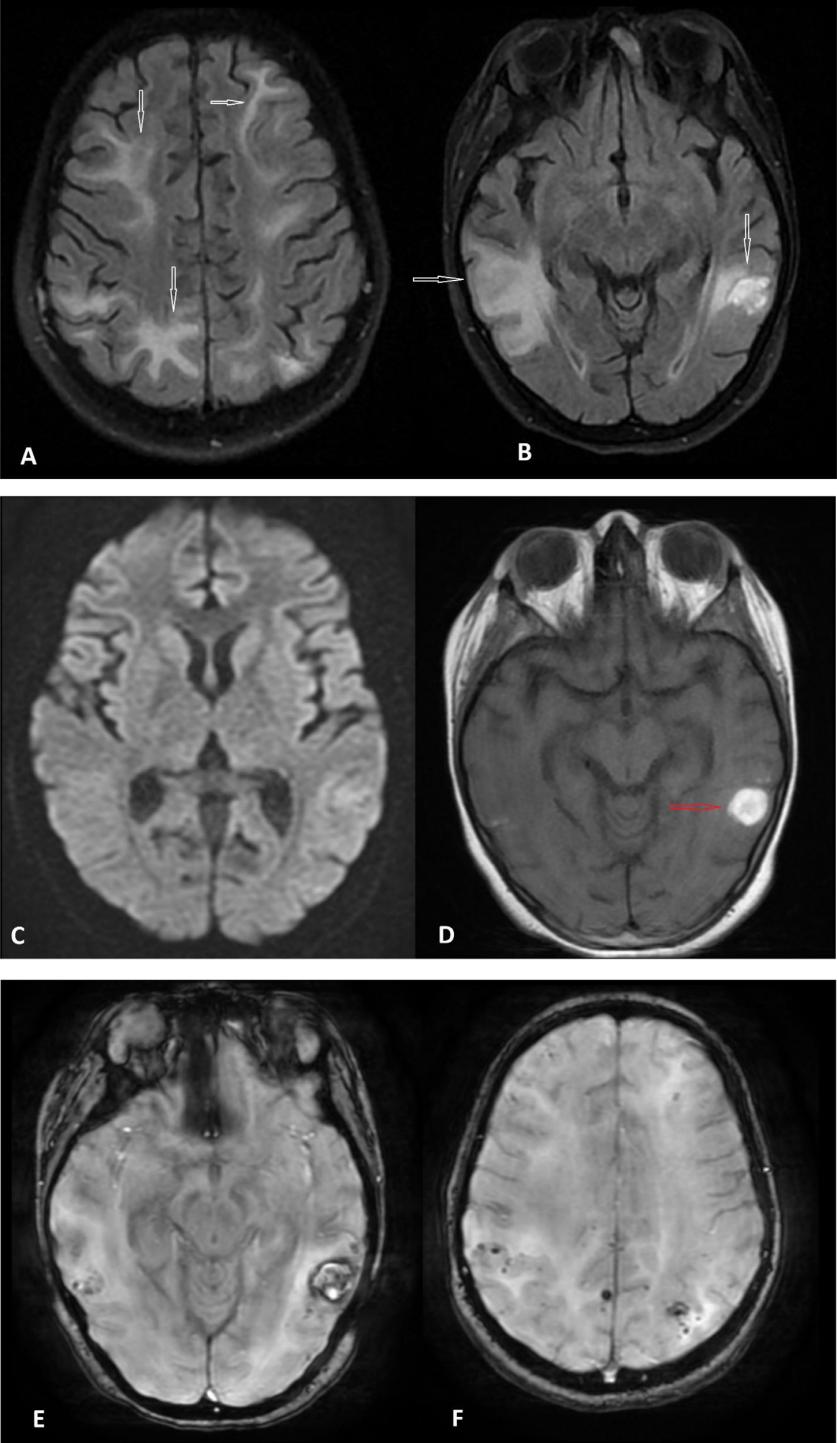
Axial plane of the brain magnetic resonance imaging without gadolinium T2/FLAIR (A, B) sequences: there are diffuse hyper signal intensities in both cerebellar hemispheres at the gray-white matter junction of the parietooccipital, frontal, and temporal lobes (white arrows). But no diffusion restriction exists on the DWI sequence (C). Hypersignal foci on T1W sequence (D) are seen (red arrow), representing hemorrhage which shows low signal intensity on SWI sequences (E, F).

The patient underwent multiplanar and multisequence imaging (MRI) and magnetic resonance venography (MRV) of the brain. The results showed small vessel vasculitis with confluent T2 hyperintensities in the gray-white matter junction of the parietooccipital and frontal lobes. Also, there was evidence of T1-weighted hypersignal foci and lesions, and the SWI sequence showed low signal intensity, which was consistent with the bleeding component (Fig. 3). No diffusion restriction was detected on DWI. These indicated PRES without edema. No signs of obvious cerebral venous sinuses or cerebral vein thrombosis were present.

After receiving prednisolone, captopril, and Rituximab effectively alleviated the individual's symptoms, stabilized vital signs, and reduced inflammatory markers. The patient was discharged from the hospital after 4 weeks and given a low-dose corticosteroid and mycophenolate mofetil maintenance medications. A 6-month follow-up examination was advised.

**Fig. 4:**
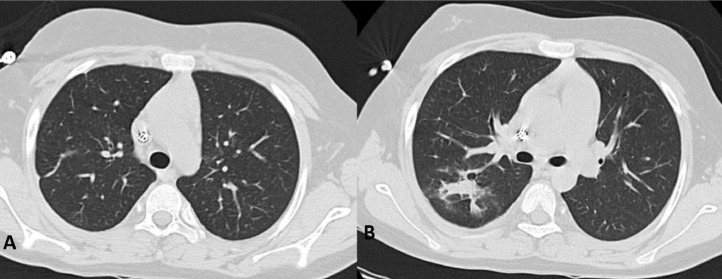
Axial (A, B) lung view of a follow-up thoracic CT scan without contrast: The previously visible cavitary lung lesions had disappeared.

The follow-up revealed that the patient had experienced significant improvement in symptoms and laboratory results. A subsequent chest CT scan also showed that the previously visible cavitary lung lesions had disappeared (Fig. 4) Furthermore, a repeat MRI confirmed that the T2-weighted hyperintense lesions had resolved. Even though the patient did not exhibit any new neurological symptoms, it was revealed that the patient was a candidate for kidney transplantation surgery due to irreversible chronic renal disease.

## Discussion and conclusion

We introduce a unique case of a pediatric patient who presented with persistent coughing, respiratory distress, and fever. Following a series of diagnostic tests, including c-ANCA, renal biopsy, and lung imaging, the patient was diagnosed with GPA. In addition to these symptoms, the patient presented with neurological manifestations and abnormal neurological exam findings. Further imaging studies, including brain MRI, revealed a rare occurrence of CNS involvement known as PRES, characterized by confluent T2 hyperintensities.  Characteristic findings may include hyperintense lesions in the parieto-occipital regions of the brain. In children, the risk factor for PRES has been linked to medical conditions such as hypertension, kidney dysfunction, organ transplantation, autoimmune or malignant disorders, and immunosuppressive treatments [9]. Several additional factors have been documented as potential causes, including Renovascular dysplasia, Pheochromocytoma, Ganglioneuroma, Primary aldosteronism, the administration of intravenous immunoglobulin, thrombotic thrombocytopenic purpura, hemolytic uremic syndrome, polyarteritis nodosa, sickle cell disease, and systemic lupus erythematosus [10,11]. While it may be rare, it is crucial to contemplate PRES as a probable cause of central nervous system symptoms in patients diagnosed with Wegener's granulomatosis. The most frequent causes of PRES in children are renal insufficiency from different sources, hematologic diseases, and their treatments, such as corticosteroid and cytotoxic agents used for malignancy or immune suppression [12].

In reviewing the literature, we found only 1 publication similar to ours, representing a 14-year-old known case of GPA with ICU admission because of PRES [9].

Akhondian J. et al. reported a rare case of PRES with spinal cord involvement (PRES-SCI) in the pediatric population. This is one of the few cases of its kind. (16) Other relevant papers discuss childhood PRES after chemotherapy for ALL as well as postoperative PRES with a sudden increase in blood pressure during recovery (15, 16) by Chaudhary et al. [12] and Rastogi et al. [13], respectively.

This study has significant strength in describing a rare case of GPA in a child with CNS involvement, known as PRES. It highlights that Wegener's granulomatosis is an infrequent cause of PRES and is even rarer in children. However, our findings should be interpreted by considering the following limitations: Unfortunately, we cannot access the patient's pathology slides from the renal biopsy as they were analyzed at a different center.

It is crucial to research GPA with PRES due to its rarity and complexity. Accurate and timely diagnosis and treatment are essential in managing this ailment to prevent organ damage.

## Patient consent

Written informed consent for publication was obtained from the patient's parents.
